# Development of a Digital Platform: A Perspective to Advance Space Telepharmacy

**DOI:** 10.1109/OJEMB.2023.3237988

**Published:** 2023-01-18

**Authors:** Marlise A dos Santos, Juliana Herbert, Ilaria Cinelli, Jose Antonio L Burmann, Vinicius V Soares, Thais Russomano

**Affiliations:** MyDigicare BrazilInnovaSpace Ltd London SE280LZ U.K.; Space & Extreme Environment Research Center, Graduate Program of Information Technology & Healthcare ManagementFederal University of Health Sciences of Porto Alegre 117303 90050-170 Porto Alegre Brazil; InnovaSpace Ltd London SE280LZ U.K.; Space & Extreme Environment Research Center, Graduate Program of Information Technology & Healthcare ManagementFederal University of Health Sciences of Porto Alegre 117303 90050-170 Porto Alegre Brazil; InnovaSpace Ltd London SE280LZ U.K.; Space & Extreme Environment Research Center, Graduate Program of Information Technology & Healthcare ManagementFederal University of Health Sciences of Porto Alegre 117303 90050-170 Porto Alegre Brazil; InnovaSpace Ltd London SE280LZ U.K.; Center for Aerospace Medicine Studies, Faculty of MedicineUniversity of Lisbon 37809 1649028 Portugal; Space & Extreme Environment Research Center, Graduate Program of Information Technology & Healthcare ManagementFederal University of Health Sciences of Porto Alegre 117303 90050-170 Porto Alegre Brazil

**Keywords:** Telepharmacy, telehealth, digital health, space pharmacy, space medicine

## Abstract

*Goal*: Lessons learned from decades of human spaceflight have helped advance the delivery of healthcare in rural and remote areas of the globe. Inclusion of the public in spaceflights is not yet accompanied by technology capable of monitoring their physical and mental health, managing clinical conditions, and rapidly identifying medical emergencies. Telepharmacy is a practice prioritizing pharmacotherapeutic guidance and monitoring to help improve patient quality of life, and can potentially expand the field of space medicine. We seek to advance pharmaceutical care through telepharmacy by developing a digital platform. *Objective:* This study focuses on the development of a digital platform for teleassistance and pharmaceutical teleconsulting services that builds on lessons learned in delivering space medicine. *Methods:* The platform contains evidence-based information on various drugs grouped by medical specialty, and also records and saves patient appointments. It has specific service protocols for service standardization, including artificial intelligence, to allow agility in services and escalation. All data is protected by privacy and professional ethics guidelines. *Results:* The telepharmacy platform is ready and currently undergoing testing for ground applications through validation studies in hospitals or medical clinics. *Conclusions:* Although developed for use on Earth, this telepharmacy platform provides a good example of how terrestrial healthcare knowledge and technology can be transferred to space missions.

## I. Introduction

Around 600 people have travelled into space since the beginning of human space exploration in 1961. The exposure of humans to the space environment clearly poses numerous challenges to astronaut health and well-being, with increased risks associated with physiological changes in microgravity, psychosocial issues in a confined spacecraft, and radiation. Collectively, these can contribute to clinical conditions, potentially leading to medical problems and emergencies. The advent of commercial spaceflights involving space tourists who are possibly less experienced and physically fit than astronauts will increase the need for greater monitoring of physical and mental health, and medical treatment and support during time spent in space. Consequently, telehealth tools and digital technology will progressively play a more critical role in monitoring and maintaining the health and well-being of astronauts and space tourists during space missions [1].

This paper presents an overview of the medical knowledge acquired during space missions over the last six decades, setting the scene for the motivation to apply a new telehealth platform to space missions, specifically in telepharmacy. The platform tries to fill a technology gap related to the medical and pharmaceutical care of astronauts during space missions and space tourists in low-Earth orbit, also considering future trips to the Moon and Mars. A digital platform associated with artificial intelligence, such as that proposed in this paper, can have multiple applications for space missions, particularly as a comprehensive and deeper understanding of space pharmacy is still lacking. This type of digital technology is designed to empower both health care providers, such as doctors and pharmacists in mission control centers, and space travelers themselves.

### A. Background - Human Health in Space

A total or partial lack of Earth's gravitational force has been shown to cause detrimental effects on human physiology. It is well known, for example, that the amount of weight bones support while in space is reduced to near zero, resulting in bone density loss. In the post-flight phase, following return to Earth, astronauts regain most, but not all, of their bone mass, especially after long-term missions [1]. Skeletal muscles, like calf muscles, are also affected, particularly those in the back and lower limbs that counteract the force of gravity. These muscles become weaker and atrophic during a space mission [2]. A typical medical condition associated with this is lumbar region back pain, which is also secondary to elongation of the spine and an increase in height during microgravity exposure [3].

Microgravity also acts upon the microbiota and immune system, which seems to become less active, potentially increasing the possibility of diseases and infections. Several skin, gastrointestinal and respiratory infections have been reported during space missions, and even the reactivation of dormant viruses [4].

The cardiovascular system redistributes blood and body fluids from the lower to upper body upon entry into space, initially increasing heart size. Subsequently, heart and plasma volumes decrease over the course of the mission. This headward shift of blood is linked to changes in vascular pressures, including intra-ocular, arterial and venous blood pressures, but is not deemed a major problem by doctors. However, spaceflight-associated neuro-ocular syndrome, known as SANS, has raised medical concerns in the last decade, as an increase in intracranial pressure constitutes a medical emergency [5], [6]. Electrocardiogram exams have demonstrated the presence of occasional premature atrial and premature ventricular contractions inflight, being more prominent during extravehicular activities. The mission of one cosmonaut was aborted due to ventricular tachycardia [7]. Although no cardiac arrest has occurred in space, several researchers have considered this possibility, conducting Cardiopulmonary Resuscitation (CPR) studies in ground-based, parabolic flight and underwater simulations of microgravity [8], [9].

A lack of adequate venous blood flow from the head and neck to the heart was recently identified in astronauts, with an internal jugular vein thrombus requiring anticoagulation, constituting a medical emergency risk of blood flow total obstruction and pulmonary embolism. Stasis of blood flow is one component of Virchow's triad that increases thrombosis risk. Ultrasound of the internal jugular veins showed gross dilation in resting astronauts during long-term missions, with an average diameter of 9.8 mm^2^ while seated on Earth, increasing to 70.3 mm^2^ in microgravity [10].

The vestibular system, located within the inner ear, is used by the body to give spatial orientation and balance. It is one of the first organs to react to microgravity exposure, with a quick and sometimes intense response, during which body spatial orientation, coordination and balance are very affected. This is known as space motion sickness, affecting 70% of astronauts in the first 72 hours of a space mission [11].

Psychological challenges include emotional issues related to isolation, confinement, social interaction and feeling homesick, which can lead to intense depression requiring medication use and psychological counselling through telemedicine. Disruption of the sleep-wake cycle can also occur, mainly secondary to alteration in the circadian rhythm [12].

Current radiation exposure limits were set by NASA in 1989 and based on a maximum 3% lifetime excess risk of cancer and neurodegenerative diseases. This risk is evaluated with a sliding scale based on age and gender, ranging from a lower career limit of 180 millisieverts (mSv) of radiation for a 30-year-old woman to an upper career limit of 700 mSv for a 60-year-old man. The radiation threshold for female astronauts was lowered by NASA, as women have more than twice the risk of developing lung cancer than men, when both genders are exposed to high levels of radiation for similar time periods. Although radiation exposure is a well-known health hazard, no medical emergency, such as radiation sickness, has been reported [13].

### B. Background - Healthcare in Space

The health and well-being of astronauts during long- and short-term missions to Earth's orbit, and nowadays of space tourists, requires constant monitoring of their physical and mental health, management of clinical conditions, and rapid identification of medical emergencies.

Consequently, the practice of space medicine relies on the Crew Health Care System, essential for ensuring crewmember health and safety, which includes: Countermeasures System; Environmental Health System; and Health Maintenance System. The medical support for upcoming missions beyond LEO is planned based on the perceived level of threat an operation poses to health or life and the identified levels of care needed. An important tool for the preservation of human health in space is the application of telehealth using telemedicine-compatible equipment, which can even allow ground-based experts to be involved in examinations. Telehealth is an area of health assistance, research, and education, in which information and telecommunication technologies are used to give support services to communities where there is a lack of healthcare or access to certain medical specialty services, as occurs in space missions. Telepharmacy is a telehealth activity in which pharmaceutical assistance is provided to remote and disadvantaged areas on Earth, as well as in extreme environments, such as space [14].

According to the World Health Organization (WHO), telepharmacy can be defined as an indispensable service in the patient-drug relationship, and the process of healing or maintenance of health can be compromised without it [15]. Telepharmacy, therefore, has the potential to improve the quality of pharmaceutical care, leading to decreases in medication errors and adverse drug-related events, while at the same time increasing the cost-effectiveness of provision and improving access to care [16]. It then becomes an essential tool for the advancement of pharmaceutical care, a practice prioritising pharmacotherapeutic guidance and monitoring to help improve patient quality of life and is of particular benefit in remote and/or disadvantaged areas, where it is often more difficult and expensive to recruit and support the activities of pharmacists [15]. Space missions take place in the extremely remote setting of space and, therefore, should be considered for Telepharmacy.

## II. Materials and Methods

The platform in Telepharmacy (Pharmaceutical Teleconsulting and Pharmaceutical Teleassistance) for use on Earth and possible application to space missions was developed over a period of one year.

The initial phase of the platform design led to the design and implementation of the system architecture, specification of its functional and non-functional requirements, and modelling of the database used. The development of the first version software system was based on features created during the initial project design. The development was planned and executed to generate executable software increments after each iteration to facilitate and accelerate the evaluation of the implemented processes, seeking greater usability, value generation and adaptation to the use context.

The system is composed of two main modules: the frontend, developed using the ReactJS library and the HTML, JavaScript and CSS languages; and the backend, developed on a server running NodeJS and the MySQL database management system.

**FIGURE 1.**
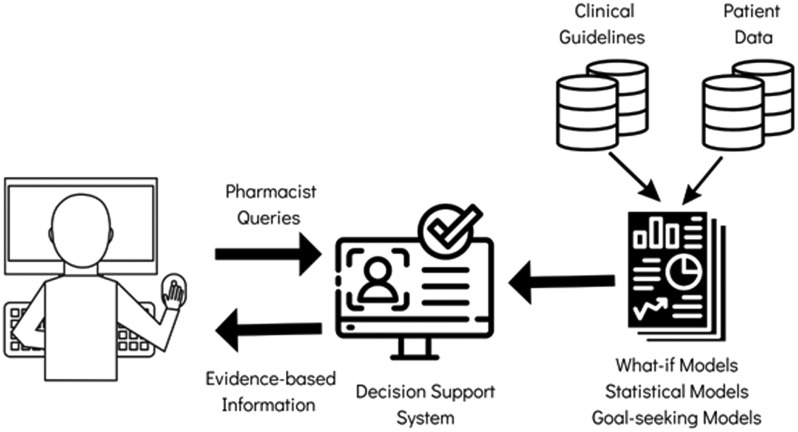
General Structure related to the Decision Support System of the Telepharmacy Platform.

The platform has specific service protocols for standardisation of service, including artificial intelligence to allow agility in services and escalation. The decision support system combines clinical guidelines approved by medical and pharmaceutical societies and patient data, suggesting actions and alerts to the pharmacist, see Fig. 1.

Its main software features include: video and audio call systems; registration of professionals, pharmacists and applicants; schedules; scheduling tele-assistance; creation of teleconsultations; professional's link to the medical record; management of professional's schedules; login; service forms for editing/viewing; informational screens/tables; website home screen; and dashboard. Data security and privacy are also incorporated in accordance with the General Data Protection.

The different platform profiles are administrator, superuser and collaborator. Each one has a specific login to access the platform. The administrator has their own email address, full access and can create, schedule, reschedule and cancel appointments, and sends the link for the patient to make the appointment. In addition to having access to administrative services, the superuser also manages the administrator. In addition to pharmacists, the platform has a nutritionist and psychologist.

### A. Operation of the Platform in Telepharmacy

The patient is referred through an attendance service, by a health professional or through the website and QR-Code. For any of these methods, the patient gets in touch by cell phone, WhatsApp or email. The administrator receives the contact and schedules an appointment, asking for the patient's name and cell phone number only, at which time a specific patient ID is created and a message, link to access the query, and a numeric code forwarded to their (or caregiver/family member) WhatsApp number.

For the consultation, the patient clicks on the link, and three numbers appear. The patient clicks on the number that corresponds to the code received. A message then appears seeking confirmation that the patient authorises the data to be collected, stored and recorded. If accepted, the patient enters the video consultation with the pharmacist, who can also see the patient on the screen.

When the link is generated by scheduling the appointment, the pharmacist who will perform the pharmaceutical appointment is selected. Thus, the consultation begins with the pharmacist collecting data/information from the patient, following protocols developed by the company, based on national and international guidelines, as well as adherence protocols.

All patient information is recorded in the consultation process. The exams and photos of medications used by the patient are sent to the pharmacist, who adds the details to a specific drive referring to the patient's chart, as well as the recordings of the consultations. The pharmacist schedules the patient's next appointment. In the case of polypharmacy and a need to start treatment with gradual doses, perform a medication withdrawal procedure, or monitor glucose and/or blood pressure, the pharmacist forwards materials to the patient as a guide to aid adherence to the medication. treatment and health monitoring. During the consultation process, the pharmacist assesses whether the patient should see another health professional, such as a doctor or nutritionist, among others. To assure suitable sensible data treatment, a specialized law professional was hired to suggest and validate data security and privacy principles were defined and implemented.

The teleconsultation begins with the medication prescriber registering on the platform, after which, a chat is initiated to request information about a drug or treatment, specifying the area. The specialist pharmacist in that area assumes the case and tries to connect with the applicant via WhatsApp. At this point, the two parties decide whether the conversation will be via video, audio or chat. The requester sends feedback citing whether the question has been resolved or not. If resolved, the case is closed, and if not, it remains open.

## III. Results

The digital telepharmacy platform was validated in two different services: pharmaceutical assistance after hospital discharge, under the multidisciplinary extension program (PROTRAC) of the Federal University of São Paulo (UNIFESP) hospital with heart disease patients; and in the Mental Health Program of the municipality of Eldorado do Sul, both are part of the Unified Health System (SUS).

A total of 20 patients with heart disease were monitored remotely by a clinical pharmacist through the platform for 30 days after hospital discharge. The protocol included 5 consultations and free WhatsApp access messages in case of emergency. The mean age of the patients was 63 ± 11 years, 60% were female and 40% were male. It is important to mention that, although all patients were classified as having heart disease, all had at least two associated comorbidities, such as systemic arterial hypertension (85% of patients), diabetes (55%) and renal failure (50% of patients). This means that all patients were considered patients of medium or high clinical complexity. Pharmaceutical interventions were required in all patients followed up. The main pharmaceutical interventions carried out were explaining the disease to the patient, changing the medication administration time, providing guidance on the use of some medications on an empty stomach, organizing the polypharmacy and the slow medication withdrawal calendars, teaching how to measure, record and monitor systemic blood pressure and glucose level, as well as the medical prescriptions. A total of 95% of the patients adhered to the treatment and 71% of the physicians accepted the suggested pharmaceutical interventions, such as changing dosage and/or medication. There was a 25% reduction in hospital readmissions, when compared to the number of readmissions before and 9 months after telepharmacy monitoring. A total of 70% of patients answered the satisfaction questionnaire and all were satisfied with the service and would recommend it to others.

Regarding the mental health program, 17 patients were followed up by a clinical pharmacist via telepharmacy for 60 days, and, according to the protocol, there was one consultation per week, in addition to free WhatsApp contact with the pharmacist in case of emergency. The mean age was 45.5 ± 13.6 years, and 65% were female and 35% male. Pharmaceutical interventions were performed in 100% of patients, which included mainly organizing the slow medication withdrawal calendar and changing the medication administration time. After 60 days of monitoring via telepharmacy, 88% of patients showed clinical improvement in symptoms and none required hospital readmission. The Morisky Medication Adherence Scale was applied at two different times: at the beginning and at the end of the follow-up period. The results showed there to be a 50% reduction in non-adherence to pharmacological treatment and a 28% increase in adherence. A total of 47% of patients answered the satisfaction questionnaire and were satisfied with the telepharmacy service.

## IV. Discussion

Human anatomy and physiology have been shaped by Earth's gravitational force. When exposed to the microgravity of space, all our cells, organs and body systems are affected. Psychosocial issues also cause concern, while space radiation is another hostile factor to which astronauts are submitted. Beyond this, there are clinical conditions and medical emergencies that crewmembers have developed during and after spaceflight. However, in general, health professionals do not always feature as members of the space crew. Telehealth, therefore, became very important, playing an essential role during space missions, even featuring in the first human spaceflight of Yuri Gagarin in 1961, when his ECG was transmitted to the mission control center on Earth [5].

The continuous advance in digital and communication technologies has progressively improved the way these systems can help monitor the well-being of astronauts during space missions and treat clinical conditions. Telemedicine is a good example of how the digital and telecommunication technologies developed for use either on Earth or in space can benefit each other. Telehealth technology applied at the International Space Station, for example, has provided inputs for the provision of healthcare in rural and remote areas of the globe. Ground-based telemedicine and digital health studies have also been conducted to evaluate how these systems could provide health assistance in the management of physical and mental issues through the evaluation of medical procedures and care of different health conditions in space missions. A complicating factor, however, is the great distances in planetary exploration, such as a trip to Mars, which will lead to time delays in communication with medical personnel on Earth, ranging from 3min to 24min each way, affecting the application of telehealth in deep space missions. This is something that does not occur on Earth, even in the most remote locations, or in missions to low-Earth orbit or the Moon [14].

The roots of telepharmacy first grew at a hospital level, through pharmaceutical care provision via telephone, with subsequent evolution as modern technologies developed, such as multimedia access tools designed to provide remote monitoring and therapy. The advent of telephone call centers also contributed to the wider use of telepharmacy, as they opened up the ability to provide medication counselling, and prior and refill authorisation for prescription drugs, as well as formulary compliance monitoring [17].

Patients with heart disease also presented important comorbidities, which required a more complex health care that negatively affected patient discharge from the hospital. Despite the interventions of the clinical pharmacist occurring weekly, the number of messages received by WhatsApp was almost double what was expected. For example - the plan for 20 patients considered a total of 100 consultations. However, an additional 83 had to be carried out, highlighting the importance of a more personalized care with human input, associated with technology, that provides a better health care. On the other hand, the reduction of hospital readmissions by 25% is an important result, as it reduces the number of patients in emergency services and costs to the SUS (Brazilian public health system). During the mental health patient follow-up program, the main problem observed in regard to treatment non-adherence was linked to the adverse effect of the medication, which is very common for this type of medication. Thus, after explaining to the patient about the treatment and the need for a gradual introduction of the medication, the level of adherence increased, according to the Morisky scale. It was also observed that many patients had social-economic problems, which included low levels of nutrients in their diet. In this circumstance, vitamins and other supplements were suggested to improve quality of life and the effectiveness of the therapeutic effect of the medication used.

Telepharmacy, therefore, has the potential to improve the quality of pharmaceutical care, leading to decreases in medication errors and adverse drug-related events, while at the same time increasing the cost-effectiveness of provision and improving access to care [15], [16].

Space pharmacy is still in its infancy, as medication dose, intake intervals, drug interactions and route of administration are based on Earth's prescriptions, and do not take into account the inevitable changes in pharmacodynamics and pharmacokinetics when the body is exposed to microgravity. Nonetheless, reports show that astronauts use medications in every space mission, with the three main types of medications used being sleeping pills (insomnia), pain killers (lumbar pain) and motion sickness drugs [18].

A telepharmacy digital platform with an integrated artificial intelligence system, such as that proposed in this paper, could play an important role and prove useful for the improvement of healthcare of astronauts and space travellers. Additionally, it can provide additional clinical information regarding drug response and effectiveness. This could be an extra tool to better understand medication distribution, absorption, metabolism, and desirable and undesirable effects, as well as drug interactions with other medications, food, supplements, radiation, and microgravity. The design of the platform is complete and operational, and it is currently undergoing validation studies on Earth. The results of these studies can be used to promote its insertion in healthcare systems and places worldwide, as well as in space missions.

## V. Conclusion

The telepharmacy platform presented in this paper was initially designed for use on Earth, targeting healthcare professionals and patients, and is especially useful in remote or deprived areas of the globe. This study has shown that a telepharmacy platform is an excellent tool to improve health care. According to our findings, however, its success depends on the combination of technology with a more humanized form of health care, which improves the comfort and safety of patients in the community, helping to reduce unnecessary visits to the emergency services and enhancing treatment adherence. Such a platform can also be applied to space travellers, whether astronauts or space tourists, on short or long-term missions in Earth orbit, or in future off-Earth bases on the Moon or Mars. A telepharmacy platform could be integrated into the telehealth system used during space missions to monitor crew health and well-being, and to provide medical assistance during emergencies and clinical situations, as the management and treatment of many diseases includes medication use.

## Authors Contribution

**Marlise dos Santos:** Conceptualization, Methodology, Investigation, Writing. **Juliana Herbert:** Methodology, Investigation, Writing, Validation. **Ilaria Cinelli - Member, IEEE**: Validation, Writing. Jose Antonio Burmann: Validation, Writing. **Vinicius Soares:** Validation, Writing. **Thais Russomano:** Methodology, Investigation, Writing, Validation.
